# Pediatric Oncology Knowledge Mobilization in Canada: Protocol for an Environmental Scan

**DOI:** 10.2196/76787

**Published:** 2026-05-01

**Authors:** Emily K Drake, Catherine Foulem, Ekaterini Damoulianos, Stephanie Reid, Patrick Cossette, Michel Duval, Kirsten Efremov, James Foster, Karen Haas, Argerie Tsimicalis

**Affiliations:** 1 Ingram School of Nursing Faculty of Medicine and Health Sciences McGill University Montréal, QC Canada; 2 Interdisciplinary Health Studies Faculty of Science Mount Allison University Sackville, NB Canada; 3 Gerald Bronfman Department of Oncology Faculty of Medicine and Health Sciences McGill University Montréal, QC Canada; 4 Department of Social Work Trent University Durham-GTA Oshawa, ON Canada; 5 Caregiver Partner Toronto, ON Canada; 6 Leucan Montréal, QC Canada; 7 Centre de Recherche Centre Hospitalier Universitaire Sainte-Justine Montréal Canada; 8 Département de Pédiatrie Université de Montréal Montréal Canada; 9 Patient Partner Toronto, ON Canada; 10 Kids Cancer Care Calgary, AB Canada; 11 Caregiver Partner Coldwater, ON Canada

**Keywords:** knowledge mobilization, cancer, children, teens, protocol

## Abstract

**Background:**

Nonprofit organizations that serve the pediatric oncology community play a crucial role in disseminating quality information that can inform and support people living with childhood cancer, those that work in the field, and others who make key decisions or policies. These registered organizations can be challenging to locate, as the internet is flux with information and resources of varying quality, misinformation, and disinformation. There remains limited understanding of the knowledge mobilization landscape of these organizations in Canada.

**Objective:**

This study will provide an overview of the pediatric oncology nonprofit organizational landscape and describe their knowledge mobilization efforts related to dissemination, highlighting existing strengths, gaps, and novel opportunities to strengthen and unite efforts.

**Methods:**

A novel environmental scan methodology will be employed to search government and nonprofit organizations’ databases. Independent reviewers will screen the websites of eligible organizations. Extracted data will be descriptively analyzed, geographically sorted, and presented in a tabular form with accompanying narrative.

**Results:**

This project received funding in 2024. We anticipate that preliminary results will be available by summer 2025. The search strategy for this study will be completed in the spring of 2025. One key project milestone for this environmental scan includes sharing drafts of the results from this strategy through expert consultations in the spring of 2025. After this milestone, a full set of preliminary results will be available by summer 2025, and the final manuscript will be submitted in fall 2025.

**Conclusions:**

The environmental scan will explicate each step of our method to allow others the opportunity to garner understanding from our learnings. Findings will be disseminated to the broader community via social media, directly to the pediatric oncology network in Canada, and globally, through summaries, infographics, presentations, and traditional academic outputs. By doing so, the pediatric oncology community will have information pertinent to navigating these resources, and further steps can be devised to bolster the knowledge mobilization capacity in Canada.

**International Registered Report Identifier (IRRID):**

DERR1-10.2196/76787

## Introduction

### Background

Each year, approximately 1000 children younger than 14 years [1] and over 400 adolescents (15-19 years of age) [2] are diagnosed with cancer in Canada. The incidence rates of pediatric cancers are slowly increasing, while the mortality rates for these illnesses continue to decrease [3]. Children and adolescents diagnosed with cancer and their families can experience a number of challenges that may include school disruptions, limitations in physical activities, and the need to navigate complex health information [4-6]. Caring for a child living with cancer can be a challenging time, and emotions may be heightened due to a lack of information or misinformation [7]. To equip the broad pediatric oncology community with needed evidence-based information, it is essential that information be shared in ways that are meaningful and accessible to community members [8].

Knowledge mobilization is the overarching term used to describe a number of different research activities that center around deliberate action to make research available and meaningful by creating relationships between knowledge users and the research team [9]. These efforts are intended to improve the quality of the research being conducted and its impact on practice and policy [9]. Part of these activities include efforts to make research findings more accessible to the broader nonacademic audience [9]. Knowledge dissemination activities involve communicating research findings by packaging tailored research results into meaningful messages for targeted audiences [10]. Access to effective evidence-based education has been shown to improve the quality of life for patients and informal caregivers and decrease stress and anxiety [11]. Broadly disseminating information has been shown to impact the knowledge to action pipeline, improving the integration of information to programs and policies [12,13].

Nongovernmental organizations (NGOs) or community-based organizations frequently fill the gaps and combat barriers to accessing vital information and support that cannot be provided for by governmental organizations [14]. In particular, organizations with mandates to serve the pediatric oncology community may be involved in the production of oncology research or serve as valuable knowledge brokers [15]. In addition to linking knowledge producers and knowledge users [16], organizations can serve as knowledge brokers through adapting evidence to unique contexts and disseminating it to their audiences [17]. However, despite the important role nonprofits may play in supporting patients and disseminating pertinent evidence-based information, there is no centralized spot where every organization supporting the pediatric oncology community is known. ACCESS (Advancing Childhood Cancer Experience, Science & Survivorship) [18], a pan-Canadian network for pediatric cancer, was formed to bring people with different roles and backgrounds together for the advancement of childhood cancer experience, science, and survivorship. However, within this network, not every nonprofit organization has been identified. In addition to this, Canada has limited knowledge of how these organizations utilize resources (eg, social media platforms, videos, webinars) for knowledge dissemination activities to inform and support those affected by childhood cancer. Uniting and understanding the key contributions of each organization will help magnify and strengthen knowledge sharing, propelling an increase in the uptake of evidence into practices and policies [9].

### Objectives

Our environmental scan will identify the knowledge mobilization efforts currently underway in Canada for the pediatric oncology community by (1) mapping a current list of nonprofits that serve the pediatric oncology community in Canada, (2) analyzing and describing the ways through which they mobilize knowledge by disseminating information, (3) identifying strengths and gaps in knowledge mobilization efforts throughout the country and potential novel opportunities to increase knowledge mobilization efforts for this population in Canada, and (4) describing a methodology for conducting an environmental scan in this research area through various internet-based sources and unique means of engaging the childhood cancer community and member checking.

## Methods

### Study Design

An environmental scan will be conducted by our research team, a group of representatives from the pediatric oncology community, including scientists, nonprofit employees, trainees, and people with lived experience, to systematically collect and analyze pediatric oncology organizations in Canada and their knowledge mobilization activities. There are no standard methodological approaches to the design and implementation of environmental scans [19-21]; therefore, our study will employ a novel environmental scan methodology (see Table 1) designed to search the internet to compile an accurate set of nonprofit organizations and knowledge mobilization efforts through a multimodal search strategy. When designing this methodology, our interdisciplinary team developed a comprehensive overview of how patients, families, and those who work in pediatric oncology may look for resources. In keeping with this, our study will involve (1) conducting a comprehensive search of government and nonprofit organizations’ databases, (2) screening results for study eligibility, (3) extracting the data, (4) conducting expert consultations, (5) generating data presentations, and (6) producing a final study report [19]. The updated framework for scoping reviews by Peters et al [22] was used to inform the development of this protocol, along with environmental scan methods identified by Castro et al [19], Choo [21], and Rowel et al [20].

**Table 1:** Methodological overview.

Step order	Step	Notes
1	Conduct a comprehensive search	Three-step bilingual search strategy will involve Searching for the nonprofit organizations identified in the development of a government-funded national pediatric oncology network and their current listed partners Searching 3 Canadian governmental databases Conducting a search of a nonprofit resource database
2	Screen results for study eligibility	Search results will be uploaded to a shared Excel document where duplicates will be removed. Each result will be visited to assess for eligibility.
3	Extract data	Each website will be saved, and a reviewer will extract information pertaining to the study eligibility criteria by using a data extraction tool
4	Conduct expert consultations	Expert consultations will be conducted through a multimodal approach
5	Data presentation	Data will be presented in a PRISMA-ScR (Preferred Items for Systematic Reviews and Meta-Analyses extension for Scoping Reviews) flow diagram, tabular forms, graphic forms, and with a narrative summary
6	Produce a final study report	None

### Eligibility Criteria

To be eligible for inclusion in this environmental scan, organizations must be a nonprofit organization. Through developing this protocol, we have seen that use of the terms nonprofit, not-for-profit, and charitable organizations are at times used interchangeably, despite their distinct definitions with the Government of Canada [23]. Although the government states that you cannot be a nonprofit and a registered charity, there are still registered charities that identify themselves as nonprofits (eg, some identify themselves as nonprofit organizations with charitable status). To recognize the use of terms both at the government and community-level, our use of the term nonprofit will be to identify organizations that ultimately have a registered charitable number.

Nonprofit organizations identified through our search strategy must meet 3 eligibility criteria. They must be (1) located in Canada, (2) disseminating information and/or resources, and (3) specifically serving the pediatric oncology community (eg, patients, caregivers, health care providers). Organizations may have missions to serve the broad pediatric cancer community or specific childhood cancers. Organizations that do not have a registered charitable number and that are designed for the broader child health community (eg, organizations that address pediatric pain) or the broader cancer community (eg, national organizations that serve all ages of cancer diagnoses) will be excluded. Those who provide information and/or resources to the adolescent and young adult cancer (15-39 years of age) [24] community will be included if their mission statement serves adolescents and targets those younger than 18 years and/or their caregivers. Similarly, organizations that serve survivors of childhood cancer who are young adults (18-39 years of age) [25] or adults will be excluded from this environmental scan. Advocacy groups that do not hold a registered charitable number and serve as social media influencers/digital opinion leaders will be excluded. Separate environmental scans will be needed for these excluded groups.

Knowledge dissemination activities of included nonprofits will be screened to descriptively analyze the efforts undertaken. Activities will be eligible for inclusion if (1) they concern a method of disseminating knowledge (eg, online, in person), (2) they are relevant to the pediatric oncology community in Canada, and (3) the content being shared or discussed involves information concerning the spectrum of cancer care from prevention to end-of-life care and bereavement.

### Data Sources and Search Strategy

#### Internet-Based Search of Nonprofit Organizational Pediatric Oncology Knowledge Mobilization Activities

Nonprofit organizational pediatric oncology activities will be identified through a 3-step bilingual search strategy in Canada’s official languages. First, Canadian pediatric oncology nonprofit organizations will be identified by (1) searching for the nonprofit organizations identified in the development of a government-funded national pediatric oncology network and their current listed partners, (2) searching 3 Canadian governmental databases, and (3) conducting a search of a nonprofit resource database. These bilingual search strategies have been developed in collaboration with people with lived experience, an expert patient/family librarian, and reviewed by consulting coauthors [19] with expertise in this research area to ensure that the keywords selected for the search strategy reflect the lens of how those with lived experience (eg, a guardian, caregiver), and/or those who advocate/work in oncology would search for informational resources regarding pediatric cancer. Keywords selected to develop the search strategies include “childhood cancer,” “pediatric cancer,” and “child cancer” (see Table 2 for the full list of our key terms and their French translation).

**Table 2:** Key search strategy words: keywords related to the study objectives and concept of pediatric oncology knowledge mobilization. English terms were translated to French by a bilingual study coauthor (ED).

Original English keywords	Translated French keywords
Childhood cancer	Cancer chez l’enfant
Pediatric cancer	Cancer pédiatrique
Pediatric	Pédiatrique
Pediatric oncology	Oncologie pédiatrique
Child cancer	Cancer de l’enfant
Adolescent cancer	Cancer de l’adolescent
AYA	AJA
Infant cancer	Cancer du nourrisson

#### Step 1: Searching Online Resources and Databases

##### Identifying ACCESS’ Nonprofit Partners

The first step in this search strategy will be to search the nonprofit organizations that have been identified through the creation of ACCESS [26]. This will include identifying those that were identified through the Canadian Institutes of Health Research’s [27] call for the Team Grant: Pediatric Cancer Consortium funding opportunity in 2022. As part of this process, we will then search the current listed partners of the ACCESS network [28].

##### Government of Canada’s Federal Corporation Database

The Government of Canada’s Federal Corporation [29] database will be searched using the key terms from Table 2 in the fillable section for corporate names. The corporation number and business number fields will remain blank. “Any” will be selected for the province of registered office, and “active” will be selected for the corporation status. “Canada Not-for-profit Corporations Act” will be selected for the governing legislation. A preliminary search of this not-for-profit database yielded organizations with registered charity numbers (eg, Lung Cancer Canada) [30].

##### Canada’s Business Registries

The Canada’s Business Registries, a federal-provincial-territorial collaborative that is supported by the Canadian Association of Corporate Law Administrators, will be searched [31]. To conduct this search, key terms from Table 2 will be entered. “All locations” will be selected for location and “All” will be selected as business type. “Active” will be selected for business status. The results will be sorted by “Best Match.” This database was chosen due to the search results yielded with charitable numbers in our preliminary search of the federal database.

##### Government of Canada’s List of Charities and Certain Other Qualified Donees

The list of charities and certain other qualified donees [32] maintained by the Canadian federal government will be searched using the basic search. For the “organization name,” each search term will be entered and searched separately. For “status,” “registered” will be selected.

##### Canadian Cancer Society’s Community Services Locator

The Canadian Cancer Society’s Community Services Locator [33] is a directory that connects caregivers, health care providers, and patients to cancer-related services. This database will be searched using the keywords in Table 2 to search for Canadian nonprofit pediatric cancer organizations; as such, no specific city or postal code will be entered into the search box. The final search results will be sorted by relevance, and no specific limitations will be applied [19].

#### Step 2: Screening

To determine the feasibility of our search strategy, we conducted preliminary searches of the Canadian Cancer Society’s Community Services Locator, the Government of Canada’s Federal Corporation database, Canada’s Business Registries database, and the federal list of charitable organizations and other qualified donees. Each preliminary search retrieved a manageable amount of hits returned for our study to be operational based on study resources.

Results will be uploaded to a shared Excel document where search results will be tracked and duplicates removed. Each result will be visited to assess for eligibility. Search results that do not meet the study’s (eg, for-profit businesses, no charity registration number, social media influencers) inclusion criteria will be excluded. Members of the team will hold discussions to determine the eligibility of results that may be unclear [19].

#### Step 3: Data Extraction

Once a nonprofit pediatric oncology organization is determined to be eligible for inclusion, each website will be saved, and a reviewer will extract information pertaining to the study eligibility criteria (see Table 3). This will include the geographic location of their headquarters; targeted audiences (eg, siblings, patients, adult survivors, caregivers, health care providers, scientists, teachers, decision makers, Indigenous community members) based on the labelling of sections of their website, if applicable; and language of their content. Data related to their knowledge mobilization efforts will be deductively extracted to capture the breadth of their knowledge dissemination strategy (eg, social media accounts, infographics, academic papers). Data extraction will be completed in a collaborative manner among the research team, with one team member leading the data extraction process. To aid in maintaining a consistent data extraction approach, a template (Table 3 and Table 4) has been created guided by the work of Cooper et al [34]. This template will serve as a working document where categories may be inductively added if there are novel ways in which organizations are mobilizing knowledge both online or through in-person offerings that our preliminary draft failed to capture. The addition of new categories will take place following discussion with the research team who will also aid in addressing discrepancies and issues that may arise throughout this process [19].

**Table 3:** Data extraction template of nonprofits headquarters, geographical location, and social media profiles.

Organization	Province/territory head office location	Provinces/territories with other locations	Year founded	Cancer type (all or specific cancers)	Audience(s)	Language(s)	Social media profiles (X, LinkedIn, Facebook, Snapchat, Instagram, Threads, Bluesky, TikTok, YouTube)
1							
2							
3							

**Table 4:** Data extraction template of the knowledge dissemination products used by the nonprofits.

Organization	Videos	Blogs	Pamphlets	Posters	Clinical practice guidelines	Online training (webinars)	In person training	Policy briefs	Academic publications
1									
2									
3									

#### Step 4: Conducting Expert Consultations

To strengthen the ability of this environmental scan to capture the breadth of pediatric cancer nonprofit organizations and their knowledge mobilization efforts, expert consultations will be conducted. Members of the ACCESS [26] network will be asked to review the list of organizations we have identified with our search strategy and to direct us to any other resources that they may know of that could meet our study’s inclusion criteria [19,35]. In addition to this group, our research team involves members of pediatric cancer nonprofit organizations and people with lived experience who will help to identify organizations and extract data, where relevant. Other stakeholders, digital opinion leaders, and identified organizations will be asked to provide feedback concerning the results and final manuscript through direct contact. Preliminary results of the included organizations will be posted on LinkedIn, Bluesky, and Instagram to ask the public to review the nonprofit organizations [19].

#### Step 5: Data Presentation

Data pertaining to the search will be presented in a PRISMA-ScR (Preferred Reporting Items for Systematic Reviews and Meta-Analyses extension for Scoping Reviews) flow diagram [36]. Data extracted from the included nonprofits’ websites will be descriptively presented in tabular and graphic forms (eg, bar charts, pie charts, scatter plots; see Table 5 for an example) that represents both the data that exist and the study’s objectives [37]. A narrative summary will accompany these graphic displays of information to illuminate for the reader how the environmental scan’s findings relate to the research objectives [37].

**Table 5:** Data presentation example.

Nonprofit characteristic	Results
Province/territory of headquarters	# by province of headquarters
Year founded	# by year
Language of content	# by languages
Audience for content	# by audience

### Ethical Considerations

This study will involve conducting a grey-literature search to identify Canadian nonprofit organizations and the different means through which they publicly mobilize knowledge to the pediatric cancer community. Ethics approval will not be required.

### Patient and Public Involvement

Four of the study’s coauthors (CF, SR, KE, and KH) are people with lived experience (eg, have been diagnosed with cancer, provided care for or were a friend to a child diagnosed with cancer). Two of our coauthors are nonprofit partners (JF and PC) representing Leucan and Kids Cancer Care. All of our patient and public partners assisted with the development of the search strategy and provided feedback on the final protocol. CF and SR helped to draft aspects of the manuscript. EKD is the cofounder of #AYACSM (Adolescent and Young Adult Cancer Societal Movement), the global adolescent and young adult cancer societal movement community [38].

## Results

This study was funded in 2024. The search strategy for this study will be completed in the spring of 2025. One key project milestone for this environmental scan includes sharing drafts of the results from this strategy through expert consultations in the spring of 2025. After this milestone, a full set of preliminary results will be available by summer 2025, and the final manuscript will be submitted in fall 2025.

## Discussion

### Overview

Conducting this environmental scan will allow us to map and examine the environmental and contextual characteristics of the nonprofits that exist in Canada to serve the pediatric oncology community. Through plotting these organizations, we will gain a richer understanding of the audiences they serve and their locations in Canada. While many organizations offer virtual offerings that serve the Canadian and broader global community, understanding where these organizations are physically situated will allow us to understand which provinces/territories do not have physical proximity to these supports. Understanding the audiences that they serve will allow us data to advocate for knowledge mobilization capacity for stakeholders that may be underserved (eg, Indigenous community members, siblings of childhood cancer survivors, newly arrived immigrants) [20]. While nonprofit organizations play a key role in knowledge brokering [15], understanding the ways through which they disseminate knowledge will allow us to develop knowledge mobilization capacity strategies pertaining to existing mediums of communication and those in need of development and innovation. Anticipated findings will raise awareness of areas for future research [20].

Environmental scans lack clear guidelines and definitions [19,20]. While this allows this type of research to be accessible and flexible, there is a need for clear guidance to assist others who would like to use this methodology to answer questions in their areas of research interest. In addition to making our research protocol accessible for others to use and adapt, we also anticipate outlining each step we take in the final research report along with any deviations to the protocol that may be necessary. While our intent is to provide a rich overview of the information that exists, it is possible that our strategy may fail to capture all the nonprofits that exist for this population in Canada. In addition, while we aim to capture all the included nonprofits’ dissemination activities, it is possible that their websites may not be up to date with their breadth of offerings.

### Dissemination

Findings from this environmental scan will allow our team to produce strategies to increase the impact and focus of ACCESS’ current knowledge mobilization efforts in Canada and globally. They will help to inform our prioritization of knowledge mobilization activities for the ACCESS network when considering audiences, languages, and mediums of content dissemination via nonprofits. These may include (1) social media communication using short videos, infographics, and text content on platforms such as Instagram, Facebook, Bluesky, and other social media platforms using hashtags and digital opinion leader accounts to enhance reach/visibility; (2) communication packages such as pamphlets, posters, newsletters, and infographics; (3) podcasts and webinars hosted with ACCESS and other partners; (4) conference presentations; and (5) open access academic knowledge dissemination of the environmental scan protocol and findings.

### Conclusion

The internet is flux with information of varying degrees of quality and educational content [39]. Nonprofit organizations can play a key role in knowledge production and brokerage. However, a comprehensive map of the nonprofit pediatric oncology organizational landscape does not exist in Canada despite the importance of these organizations in knowledge mobilization. This environmental scan will shed light on the characteristics of these nonprofit organizations. By identifying their means of disseminating knowledge, we will be able to map resources that exist alongside highlighting gaps and opportunities to be considered when developing the knowledge mobilization capacity for our country. The methodology outlined in this protocol will allow us to address our study’s research objectives while also explicating steps that others can take should they wish to adopt our methodology for their environmental scan context.

## Abbreviations

ACCESS: Advancing Childhood Cancer Experience, Science & Survivorship
AYACSM: Adolescent and Young Adult Cancer Societal Movement
PRISMA-ScR: Preferred Items for Systematic Reviews and Meta-Analyses extension for Scoping Reviews

## Data Availability

All data from this environmental scan will be made available through publication via tables, figures, and a description of the results.

## References

[1] Ellison L, Xie L, Sung L (2021). Trends in paediatric cancer survival in Canada, 1992 to 2017. Health Rep.

[2] Fernandez C, Fraser GAM, Freeman C, Grunfeld E, Gupta A, Mery LS, De Pauw Sonja, Schacter B (2011). Principles and recommendations for the provision of healthcare in Canada to adolescent and young adult-aged cancer patients and survivors. J Adolesc Young Adult Oncol.

[3] Xie L, Onysko J, Morrison H (2018). Childhood cancer incidence in Canada: demographic and geographic variation of temporal trends (1992-2010). Health Promot Chronic Dis Prev Can.

[4] Kuntz N, Anazodo A, Bowden V, Sender L, Morgan H (2019). Pediatric cancer patients' treatment journey: child, adolescent, and young adult cancer narratives. J Pediatr Nurs.

[5] Bryan G, Kelly P, Chesters H, Franklin J, Griffiths H, Langton L, Langton L, Wakefield CE, Gibson F (2021). Access to and experience of education for children and adolescents with cancer: a scoping review protocol. Syst Rev.

[6] Maurer SH, Hinds PS, Spunt SL, Furman WL, Kane JR, Baker JN (2010). Decision making by parents of children with incurable cancer who opt for enrollment on a phase I trial compared with choosing a do not resuscitate/terminal care option. JCO.

[7] Gurtovenko K, Fladeboe KM, Galtieri LR, King K, Friedman D, Compas B, Breiger D, Lengua L, Keim M, Kawamura J, Katz LF (2021). Stress and psychological adjustment in caregivers of children with cancer. Health Psychol.

[8] Ross-Hellauer T, Tennant JP, Banelytė V, Gorogh E, Luzi D, Kraker P, Pisacane L, Ruggieri R, Sifacaki E, Vignoli M (2020). Ten simple rules for innovative dissemination of research. PLoS Comput Biol.

[9] Knowledge mobilisation framework. The Australian Prevention Partnership Centre.

[10] Government of Canada (2010). CE handbook - chapter 7: knowledge dissemination and public outreach (focus area 4). Canadian Institutes of Health Research.

[11] Rodgers CC, Laing CM, Herring RA, Tena N, Leonardelli A, Hockenberry M, Hendricks-Ferguson V (2016). Understanding effective delivery of patient and family education in pediatric oncology. J Pediatr Oncol Nurs.

[12] Voll M, Fairclough DL, Morrato EH, McNeal DM, Embry L, Pelletier W, Noll RB, Sahler OJZ (2022). Dissemination of an evidence-based behavioral intervention to alleviate distress in caregivers of children recently diagnosed with cancer: Bright IDEAS. Pediatr Blood Cancer.

[13] Golhasany H, Harvey B (2023). Capacity development for knowledge mobilization: a scoping review of the concepts and practices. Humanit Soc Sci Commun.

[14] Nordin N, Khatibi A, Azam SMF (2022). Nonprofit capacity and social performance: mapping the field and future directions. Manag Rev Q.

[15] Meyer M (2010). The rise of the knowledge broker. Science Communication.

[16] McCall J, Mollison A, Browne A, Parker J, Pauly B (2017). The role of knowledge brokers: lessons from a community based research study of cultural safety in relation to people who use drugs. CJAR.

[17] Government of Canada (2010). Knowledge translation in health care. Canadian Institutes of Health Research.

[18] About us. ACCESS.

[19] Castro A, Lalonde-LeBlond G, Freitas Z, Arnaert A, Bitzas V, Kildea J, Moffatt K, Phillips D, Wiseblatt L, Hall A, Després Véronique, Tsimicalis A (2024). In-home respite care services available to families with palliative care needs in Quebec: novel digital environmental scan. JMIR Nurs.

[20] Rowel R, Moore N, Nowrojee S, Memiah Peter, Bronner Yvonne (2005). The utility of the environmental scan for public health practice: lessons from an urban program to increase cancer screening. J Natl Med Assoc.

[21] Choo C (2001). Environmental scanning as information seeking and organizational learning. Information Research.

[22] Peters MDJ, Marnie C, Tricco AC, Pollock D, Munn Z, Alexander L, McInerney P, Godfrey CM, Khalil H (2020). Updated methodological guidance for the conduct of scoping reviews. JBI Evid Synth.

[23] Government of Canada (2016). Differences between a registered charity and a non-profit organization.

[24] (2017). Adolescents & Young Adults With Cancer: A System Performance Report.

[25] Editors (2011). What should the age range be for AYA oncology?. J Adolesc Young Adult Oncol.

[26] ACCESS Advancing Childhood Cancer Experience, Science & Survivorship.

[27] Government of Canada Canadian Institutes of Health Research.

[28] ACCESS Our partners.

[29] Government of Canada (2025). Search for a federal corporation.

[30] Donate now. Lung Cancer Canada.

[31] Canada's Business Registries.

[32] Government of Canada List of charities and certain other qualified donees - basic search. Canada Revenue Agency.

[33] Community services locator. Canadian Cancer Society.

[34] Cooper A, Rodway J, Read R (2018). Knowledge mobilization practices of educational researchers across Canada. CJHE.

[35] Légaré France, Politi MC, Drolet R, Desroches S, Stacey D, Bekker H, SDM-CPD Team (2012). Training health professionals in shared decision-making: an international environmental scan. Patient Educ Couns.

[36] Tricco AC, Lillie E, Zarin W, O'Brien KK, Colquhoun H, Levac D, Moher D, Peters MD, Horsley T, Weeks L, Hempel S, Akl EA, Chang C, McGowan J, Stewart L, Hartling L, Aldcroft A, Wilson MG, Garritty C, Lewin S, Godfrey CM, Macdonald MT, Langlois EV, Soares-Weiser K, Moriarty J, Clifford T, Tunçalp Özge, Straus SE (2018). PRISMA Extension for Scoping Reviews (PRISMA-ScR): checklist and explanation. Ann Intern Med.

[37] Drake EK, Weeks L, van Manen Michael, Curran Janet, McKibbon Shelley (2021). The delivery of palliative and end-of-life care to adolescents and young adults living with cancer: a scoping review protocol. JBI Evid Synth.

[38] Drake EK, Weeks LE, van Manen M, Taylor D, Ricci I, Curran J (2025). How advocates can support young adults living with cancer and their transition to palliative care. Qual Health Res.

[39] Teplinsky E, Ponce SB, Drake EK, Garcia AM, Loeb S, van Londen G, Teoh D, Thompson M, Schapira L (2022). Online medical misinformation in cancer: distinguishing fact from fiction. JCO Oncology Practice.

